# Smaller Absolute Quantities but Greater Relative Densities of Microvessels Are Associated with Cerebellar Degeneration in Lurcher Mice

**DOI:** 10.3389/fnana.2016.00035

**Published:** 2016-04-19

**Authors:** Yaroslav Kolinko, Jan Cendelin, Milena Kralickova, Zbynek Tonar

**Affiliations:** Biomedical Center, Faculty of Medicine in Pilsen, Charles University in PraguePilsen, Czech Republic

**Keywords:** blood microvessels, cerebellum, cerebral degeneration, mice, laminin, Lurcher, stereology, quantitative histology

## Abstract

Degenerative affections of nerve tissues are often accompanied by changes of vascularization. In this regard, not much is known about hereditary cerebellar degeneration. In this study, we compared the vascularity of the individual cerebellar components and the mesencephalon of 3-month-old wild type mice (*n* = 5) and Lurcher mutant mice, which represent a model of hereditary olivocerebellar degeneration (*n* = 5). Paraformaldehyde-fixed brains were processed into 18-μm thick serial sections with random orientation. Microvessels were visualized using polyclonal rabbit anti-laminin antibodies. Then, the stacks comprised of three 5-μm thick optical sections were recorded using systematic uniform random sampling. Stereological assessment was conducted based on photo-documentation. We found that each of the cerebellar components has its own features of vascularity. The greatest number and length of vessels were found in the granular layer; the number of vessels was lower in the molecular layer, and the lowest number of vessels was observed in the cerebellar nuclei corresponding with their low volume. Nevertheless, the nuclei had the greatest density of blood vessels. The reduction of cerebellum volume in the Lurcher mice was accompanied by a reduction in vascularization in the individual cerebellar components, mainly in the cortex. Moreover, despite the lower density of microvessels in the Lurcher mice compared with the wild type mice, the relative density of microvessels in the cerebellar cortex and nuclei was greater in Lurcher mice. The complete primary morphometric data, in the form of continuous variables, is included as a supplement. Mapping of the cerebellar and midbrain microvessels has explanatory potential for studies using mouse models of neurodegeneration.

## Introduction

Neurodegenerative diseases are often accompanied by microvascular changes in the affected tissue that in some cases are just secondary, while in other cases they may play a distinct role in the pathogenesis of the degeneration (Kolinko et al., 2015). Furthermore, the abnormal density of microvessels might positively or negatively influence the regenerative potential of the tissue and may also potentially influence the treatment efficiency. Vascularization of the tissue is one of the most important factors determining features of the niche that might be of importance, for instance, for the development and functional integration of neural and stem cell grafts (Cendelin, 2016). In this regard, almost nothing is known about the vascular changes in cerebellar degeneration. Hereditary cerebellar ataxias represent a wide and heterogeneous group of neurodegenerative diseases that are relatively rare but usually have a detrimental impact on the patient's motor functions as well as the patient's cognitive and emotional processes (Manto, 2005).

Information about cerebellar microcirculation is limited. The development of the hindbrain (the cerebellum and the pons) and the midbrain from the metencephalon in mice occurs approximately on embryonic day 8 (Millet et al., 1996; Butts et al., 2014). Simultaneously, the superior cerebellar artery develops as a branch of the future basilar artery and is the first blood source of the cerebellum. Later, the vertebral arteries are joined (Burger et al., 2007). Three branches of the basilar artery fit directly into the cerebellum. The general principles of the organization of the microvascular network have been described only in tortoises (Kleiter and Lametschwandtner, 1995). The short arteries of the second order (A2) and the recurrent branches from A3 arterioles supply the capillary bed of the sub-pial molecular layer, while the V2 and V3 venules drain the capillary bed of the molecular layer. The dense granular layer is supplied by A4 arterioles, those adjacent to the Purkinje cell layer, and also by A3 arterioles. The areas supplied by A4 arterioles are drained via V4 venules. The Purkinje cells are vascularized by horizontal branches (“parallel arteries”) of the A4 and A3 arterioles, which capillarize toward the granular and molecular layers (Kleiter and Lametschwandtner, 1995). The microcirculation of the mouse cerebellum seems to be similarly organized.

In addition, the Purkinje cells obtain nutrients from the vessels of both adjacent layers. This means that the molecular and the granular layers have circulatory beds that are largely independent from each other. Moreover, the capillary pericytes are able to increase blood flow and correct neural activity (Hall et al., 2014). Sex differences in some features of brain vascularization have been described previously (Huang et al., 2013).

Most previous researchers have studied exercise-related capillary changes in the motor cortex and the cerebellum (Isaacs et al., 1992; Kleim et al., 2002; Swain et al., 2003). It has been shown that capillary growth occurs in the motor areas of the cortex as a robust adaptation to prolonged motor activity. However, a quantitative analysis of the vascularization of individual components of the cerebellum has not been provided yet.

Mutant or transgenic mouse models have been used to investigate the features, pathogenesis and therapeutic approaches for hereditary cerebellar ataxias (Cendelin, 2014). The Lurcher mouse is one of the most well-known spontaneous mutant models of olivocerebelar degeneration (Phillips, 1960; Porras-García et al., 2013). It is caused by a mutation (*Grid2*^Lc^) in the gene encoding the delta 2 glutamate receptor (GluRδ2) that is predominantly localized on the dendrites of the Purkinje cells and on several hindbrain neurons (Araki et al., 1993; Lomeli et al., 1993; Takayama et al., 1995). Heterozygous Lurcher mice (Lc) suffer from a complete loss of Purkinje cells within 3 months of age due to chronic depolarization of the cell membrane (Zuo et al., 1997). The activation of the intracellular cell death enzymes promotes the excitotoxic neural degeneration by launching an extracellular chain reaction of the tissue proteases (Lu and Tsirka, 2002). Loss of the Purkinje cells is accompanied by fast secondary (target-related) degeneration of 90% of the granule cells and ~70% of the inferior olive neurons (Caddy and Biscoe, 1979; Wetts and Herrup, 1982a,b; Heckroth and Eisenman, 1991; Wullner et al., 1995; Doughty et al., 2000) as well as a massive loss of inhibitory interneurons of the cerebellar cortex (Zanjani et al., 2006). Cerebellar nuclei have been reported to undergo relatively mild degeneration (Heckroth, 1994a,b). Neuron loss triggers a typical, but abnormally persistent, inflammatory reaction in the Lurcher mutant cerebellum (Vernet-der Garabedian et al., 2013). Cerebellar degeneration results in ataxia (Fortier et al., 1987) as well as various cognitive and behavioral abnormalities (Lalonde et al., 1988; Hilber et al., 2004; Cendelin et al., 2014; Lorivel et al., 2014; Tuma et al., 2015) in heterozygous Lurcher mice. Trophic support of damaged tissue through the host vascular bed is the simplest way for the correction and minimizing of the effects of the degeneration (Cendelin, 2016).

Homozygosity of the *Grid2*^*L*^c mutation causes a massive loss of the hindbrain neurons during embryonic development, and large neurons are completely absent after birth (Cheng and Heintz, 1997). Such massive degeneration leads to death at an early age (Cheng and Heintz, 1997; Resibois et al., 1997). Therefore, homozygous Lurcher mice are not convenient to use for long-term experiments. The wild type littermates of the Lurcher mutants are completely healthy and serve as optimal controls.

The microvascular network of the mouse cerebellum has not been described in detail yet and very little is known about the changes that accompany the degenerative effects of the cerebellum in Lurcher mice. Therefore, we aimed to perform a stereological study of the microvessel network of the mouse cerebellum and the mesencephalon, which is a structure topographically and, from the embryological point of view, close to the cerebellum and which has not been reported to be changed in Lurcher mice. Particular aims were to compare the capillary network in healthy and Lurcher mutant mice, to characterize the distribution of the capillaries between individual components of the cerebellum. For these purposes we estimate the total number and length of vessels, the numerical and length density of the microvascular network, and the mean length and diffusion distances of vessels. To address these questions, the following null hypotheses were formulated and tested:

1. The total number and length of the microvessels, the numerical and length density of the vessels, and the mean length, and diffusion distance of vessels are the same in the specified cerebellar structures.
2. There are no differences in the above mentioned particular parameters between the wild type and the Lc mice.
3. The correlations among the morphometric characteristics of the individual cerebellar structures and the midbrain are the same in the wild type and the Lc mice.

## Materials and methods

### Animals

Three-month-old male Lc (*n* = 5) mice and healthy wild type (WT, *n* = 5) mice from identical litters of the B6CBA strain were used. The mice were kindly provided by Prof. A. Resibois from the Université Libre de Bruxelles, and the mice for the experiments were then generated by crossing wild type females and heterozygous Lurcher males in the breeding facility of the Faculty of Medicine in Pilsen.

All of the mice were housed in plastic cages (22 × 25 × 14 cm), 2–4 mice per cage, in the same room of the breeding facility under identical conditions. The mice were kept in a room with controlled temperature (22–23°C) and humidity (50–60%), 12/12 h light/dark cycle (6 a.m.–6 p.m.). Food (standard commercial pellet diet) and water were available *ad libitum*.

All of the experiments reported here were conducted in full compliance with the EU Guidelines for scientific experimentation on animals and with the permission of the Ethics Commission of the Faculty of Medicine in Pilsen. All efforts were made to minimize discomfort.

### Processing of samples and tissue sections

The brains, after decapitation, were fixed in 10% phosphate-buffered formalin and stored in the paraffin blocks. The blocks were processed into 18-μm thick systematic serial sections (288 ± 28 sections per animal) using a microtome Leica RM 2145. The cutting plane of each sample was randomized using the orientator (Mattfeldt et al., 1990; Nyengaard and Gundersen, 1991; Huang et al., 2013). The sections were mounted onto slides, three sections per slide. Then, every fifth slide was stained with the antibodies and colored by hematoxylin. The stained sections were used for the stereological analysis.

### Immunohistochemistry

Microvessels in the mouse cerebellum and midbrain were visualized using the polyclonal rabbit anti-laminin antibody (dilution 1:50; Dako, Glostrup, Denmark, No. Z009701). The negative immunohistochemistry controls were performed according to manufacturer recommendations. The mouse colon was used for the positive control.

The sections were dewaxed in xylene, rehydrated, and successively incubated in (1) cooled acetone for 10 min, (2) 1% Normal Goat Serum for 10 min at room temperature, (3) primary antibody solution for 12 h at 4°C, (4) N-Histofine Simple Stain MAX PO (Multi, Nichirei biosciences Inc.) for 30 min at room temperature, and (5) Liquid DAB+ Substrate Chromogen System (Dako, DAB Chromogen) for 1–4 min visualization.

Then, the standard procedure for the visualization of nuclei using Goll's hematoxylin was performed, and the sections were dehydrated by alcohol, cleared using xylene, stained in a solakryl mounting medium and covered.

### Image acquisition and processing

**Figure 1:**
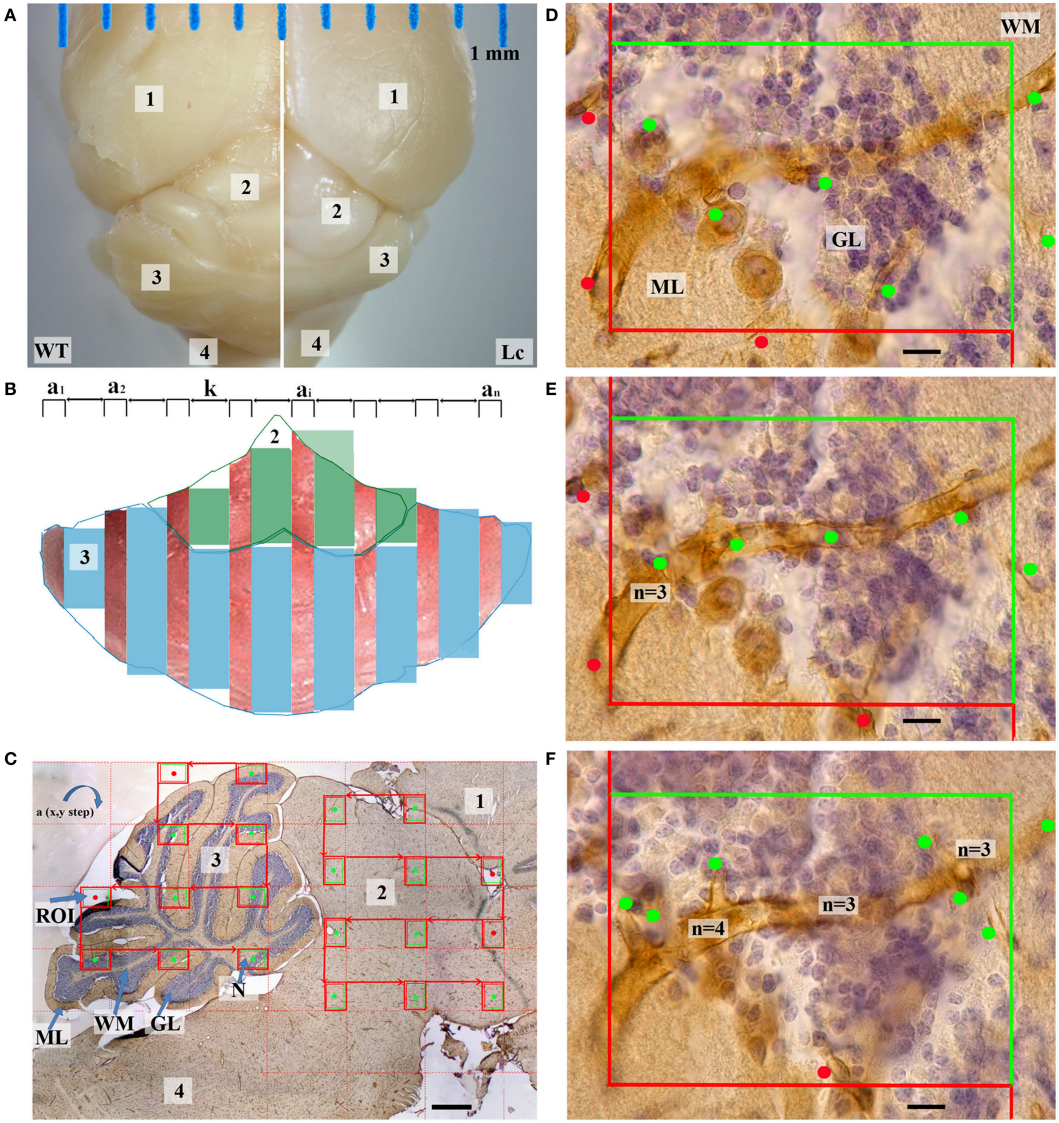
**Quantification of cerebellar and midbrain microvessels. (A)** —The dorsal view of the wild type (WT) and Lurcher (Lc) fixed brain contained the following parts: cerebral cortex (1), midbrain (2), cerebellum (3), pons and medulla (4); **(B)**—The preparations were processed into serial of equidistant randomly orientated sections (a1, a2, …ai …an) with constant step k; **(C)**—Sagittal section through the midbrain and cerebellum: nuclei (N), white matter (WM), granular (GL), and molecular layers (ML) of the cerebellum. The microscopic regions of interest (ROI) in the x–y plane were selected at a constant interval. This was performed for the cerebellum and midbrain separately. Only fields marked with the green dots were taken into account; **(D–F)**—One of the microscopic fields is shown as a stack with three 5 μm-thick optical sections in the z-axis that illustrates a disector volume probe. Vessel profiles (marked by dots) and valence of nodes (n) are marked with respective dots. Scale bars: **(C)** 500 μm; **(D–F)** 10 μm.

For imaging and further analysis, one brain section from each prepared glass (16 ± 2 sections per unit) was used. It was photo-documented in accordance with the systematic sampling rules (West et al., 1991; Burke et al., 2009; Boyce et al., 2010). In the first phase, a full section was imaged using a Plan N 2 × microscope objective with a numerical aperture of 0.06 to quantify the volume of the specified cerebellum layers and the midbrain (Figures 1B–C).

In the second phase, the section was recorded using the high numerical aperture oil immersion objective (UPpanSApo 60x/1.35 oil). From the randomly located first probe each third microscopic imaging field in the x–y plane was selected (Figure 1C; Nyengaard and Gundersen, 2006). The images of the cerebellum and the midbrain were made separately. Each microscopic field was prepared as three 5 μm-thick optical sections in the z-axis (Figures 1D–F) that illustrated a disector volume probe (Sterio, 1984; West et al., 1991) (2600 ± 702 images per animal). These techniques have been described in more detail previously (Kolinko et al., 2015). The brain stereotaxic atlas (Franklin and Paxinos, 2008) was used to identify separate parts of the mouse brain in each of the sections.

### Methods of obtaining and analyzing data

Because the Purkinje cells do not have their own microvascular supply (Kleiter and Lametschwandtner, 1995), for convenience, we decided to combine them with one of the cortex layers. The Purkinje cell dendrites are situated in the molecular layer (Caddy and Herrup, 1990; Butts et al., 2014), and this was the main reason for combining them with the molecular layer. Furthermore, their coloration was another reason (Figures 1, **4**).

The vascularity of the mouse cerebellum was characterized separately into the following parts: the molecular layer that includes the Purkinje cells, the granular layer, the white matter, and the nuclei. The vascularity of the whole midbrain was examined.

For a quantitative analysis, the histological parameters listed in Table 1 were used.

**Table 1:** **Quantitative parameters used for morphometry of vascularity in the cerebellum and midbrain, their stereological principles, histological staining, and sampling of photographs**.

**Abbreviation**	**Parameter (unit)**	**Stereological principle used for quantification**	**Objective magn**.
*V*	Volume of individual cerebellum layer, all cerebellum or midbrain (mm^3^)	Step 1. Systematic uniform random sampling of microscopic image fields selected for quantification from multiple physical sections.	2x
		Step 2. Point grid and Cavalieri of Delesse principle.	
*N*	Total number of microvessels	Step 1. Systematic uniform random sampling of microscopic image fields selected for quantification from multiple physical and optical sections.	60x
		Step 2. Disector volume probe.	
*N_*D*_*	Numerical density of vessels per unit volume (mm^−3^)	Step 1. Systematic uniform random sampling of microscopic image fields selected for quantification from multiple physical and optical sections.	60x
		Step 2. Disector volume probe.	
*L*	Total length of microvessels (mm)	Step 1. Systematic uniform random sampling of microscopic image fields selected for quantification from multiple physical and optical sections.	60x
		Step 2. Counting frame.	
*L_*D*_*	Length density of vessels per unit volume (mm^−2^)	Step 1. Systematic uniform random sampling of microscopic image fields selected for quantification from multiple physical and optical sections.	60x
		Step 2. Disector volume probe.	
*D_*D*_*	Diffusion distance of vessels (mm)	Step 1. Determinations the *L_*D*_*	–
		Step 2. Calculation according specific equation (Isaacs et al., 1992).	

*To maximize the reference space for each parameter, the lowest possible magnification was used that provided a resolution that guaranteed reliable visual control of the structures under study*.

The object *V* was quantified according to the following equation:

$$\begin{array}{l} V=h\cdot \left(k\cdot \sum {a-a}_{max}\right) \end{array}$$

where Σ*a* is the sum of the area of the sections, *h* is the height of these sections, *k* is a constant step between the sections sampled (in our case 15) and for smoothing the volume between the sections (Figure 1B) the largest section in area (*a*_*max*_) was excluded from the estimation (Gundersen, 1986).

The coefficient of error (CE) was estimated using the method proposed by Gundersen and Jensen (1987). As the first step, three sums of section areas were calculated:

$A=\sum a_{i}^{2}$ - taking into account all sections selected from the first (*a*_1_) to the last (*a*_*n*_);
*B* = ∑*a*_*i*_ · *a*_*i*+1_ - taking into account all sections selected from a_1_ to a_n−1_;
*C* = ∑ *a*_*i*_ · *a*_*i*+2_ - taking into account all sections selected from a_1_ to a_n−2_.

Then, the CE was calculated using the following relationship:

$$\begin{array}{l} \text{CE}=\frac{\sqrt{\frac{3\text{A}+\text{C}-4\text{B}}{12}}}{\sum {\text{a}}_{\text{i}}} \end{array}$$

The *N, N*_*D*_*, L*, and *L*_*D*_ were estimated using an optical disector probe (Sterio, 1984; Gundersen, 1986; West et al., 1991). Briefly, the structures were counted in the small (reference) volume samples and then, proportionally, the values for the total object volume were determined. If selected regions of interest (ROI) contained more than one layer then each cerebellar part was counted into the relevant ROI using the point grid method (Howard and Reed, 1998).

For the *N* estimation, the vessels were considered a node-segment network where the node is a place where the blood vessel branches. Consequently, a microvessel was defined as a loop created between two nodes of the vascular network. To estimate the number of loops, we determined the number of network nodes. The number of nodes was determined using the Cavalieri principle (Nyengaard and Gundersen, 2006). Briefly, an unbiased counting frame was randomly positioned upon the thick images and the captured microvessel nodes were counted (Figures 1D–F). The number of loops in the reference space was indirectly estimated in the following manner (Løkkegaard et al., 2001; Tonar et al., 2011):

$$\begin{array}{l} N_{ref}=\sum \left(\frac{\text{n}-2}{2}*{\text{P}}_{\text{n}}\right)+1 \end{array}$$

where *N*_*ref*_ is the vessel number per reference volume and *P*_*n*_ is the node number of a valence *n*. The valence of a node depends on the number of vessel segments that join at the node.

The *L* was estimated by determining the probability of the vessels being intersected by the equidistant optical slices. In this technique, *L* was equal to double the number of the vessel profile per section area (the green dot in Figures 1D–F) (Smith and Guttman, 1953; Mayhew, 2005; Eržen et al., 2011; Mühlfeld et al., 2013) taking into account all marked pieces of the vessels, regardless of form or size. When the microvessel profiles were positioned exactly on the border between two layers, the upper left margin of the profile was taken as the decisive point consistently throughout the whole study.

When the *N* and the *L* are calculated, the mean length of a single vessel as well as the distance to which the vessel is potentially capable of spreading nutrients are easily determined. Because the contour of the vascular diffusion, relative to the center of the vessel, is not uniform, the diffusion distance (*D*_*D*_) of a single vessel was determined using the following equation:

$$\begin{array}{l} D_{D}=\frac{\Gamma \left(1+\frac{1}{2}\right)}{{\pi L_{D}}^{1/2}} \end{array}$$

where Γ is the gamma function (Isaacs et al., 1992). This means that the *D*_*D*_ is inversely proportional to the square root of the density of the blood vessels.

Primary data were generated using the stereological software Ellipse version 2.0.8.1 (Vidito, Kosice, Slovak Republic). Statistical conclusions were determined in StatSoft Statistica 10 (Dell Software). For planned comparison we used the Mann–Whitney U-test to compare both animal groups. The Wilcoxon signed-rank test was used for comparing the data within each group. Spearman's coefficient was used to show the correlation between two parameters.

## Results

### The structural features of cerebellar vascularity in wild type mice

**Figure 2:**
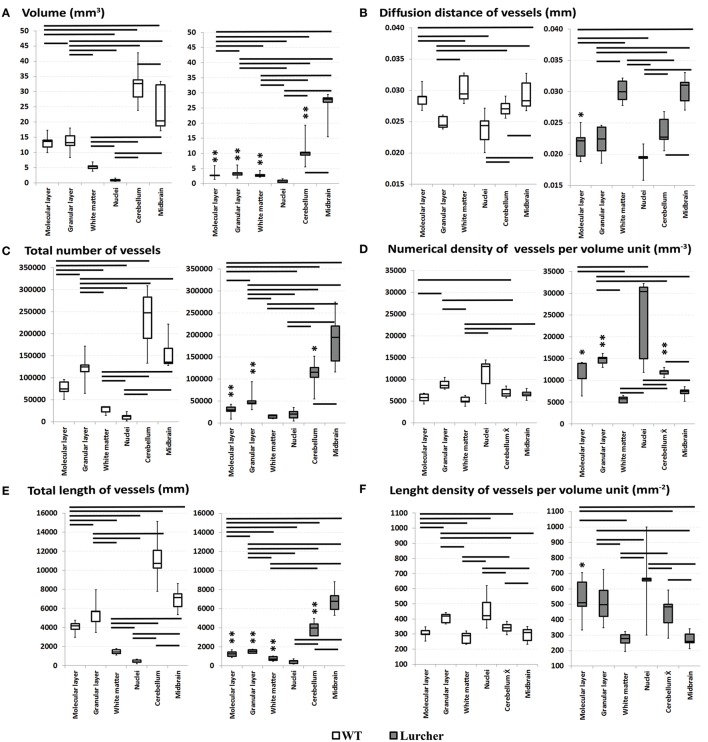
**Comparing quantitative parameters of the microvascular bed of midbrain and cerebellar anatomical compartments in wild type mice (left) and Lurcher mice (right)**. **(A)**—Volume of the cerebellum, its individual parts and the volume of the midbrain; **(B)**—Diffusion distance of vessels; **(C)**—Total number of vessels; **(D)**—Numerical density of vessels; **(E)**—Total length of vessels; **(F)**—Length density of vessels. Significant results of the Wilcoxon matched pairs test within group are connected with lines (*p* < 0.05). Corresponding anatomical compartments between groups were compared using the Mann-Whitney U test (significant results are presented within the diagrams: ^*^p < 0.05, ^**^p < 0.01).

**Figure 3:**
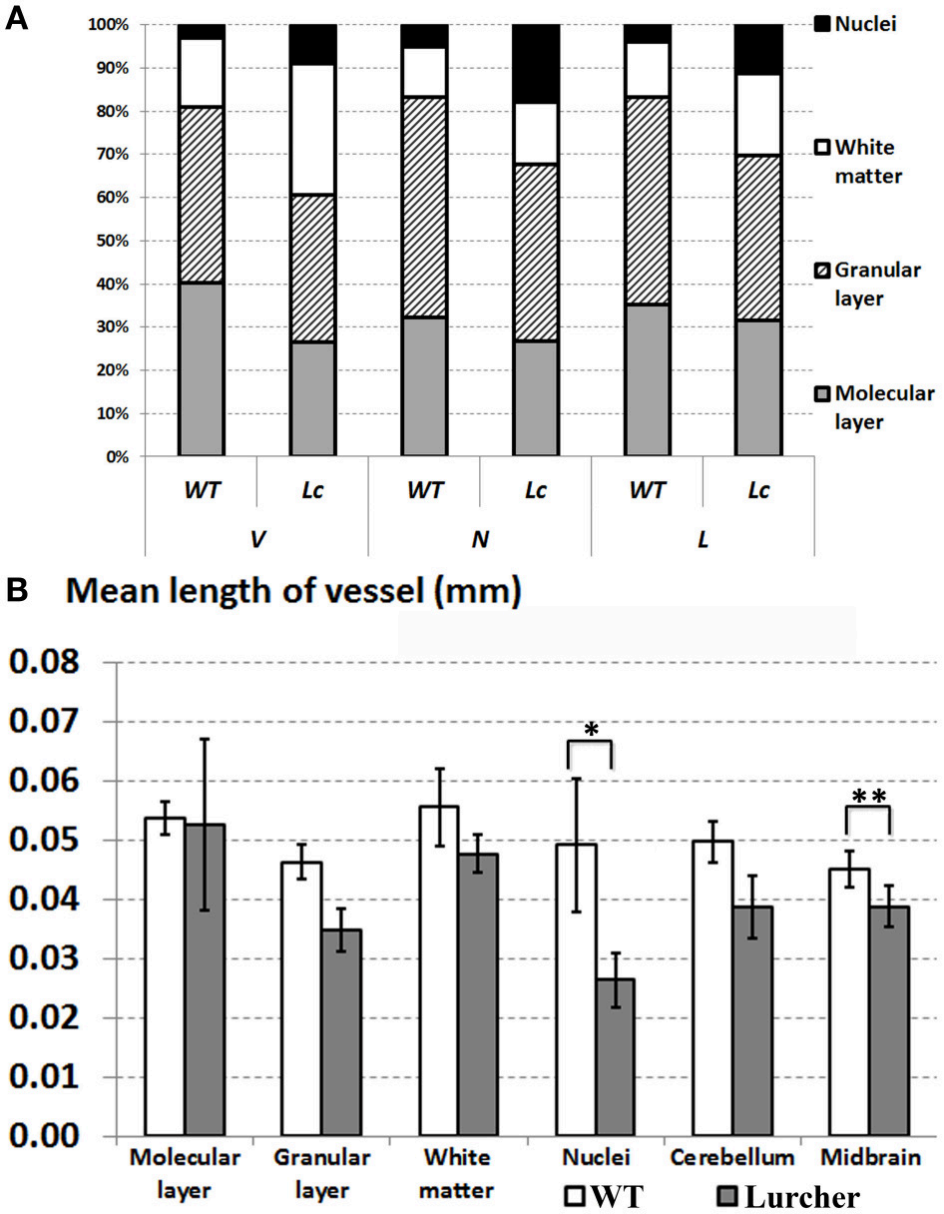
**Comparing quantitative parameters of microvessels between wild type (WT mice) and Lurcher mice**. **(A)**—Relative proportions of the individual components of the cerebellum; **(B)**—Mean length of vessels in the cerebellum, its individual components and in the midbrain. Corresponding anatomical compartments were compared using the Mann-Whitney U test (significant results are presented within the diagrams: ^*^p < 0.05, ^**^p < 0.01).

The volume of the cerebellum, as well as its individual parts, and the volume of the midbrain are shown on Figure 2A. The relative proportions of the individual components of the cerebellum are shown in Figure 3A. The volume of the cerebellum in WT mice exceeded the volume of the midbrain. The largest share of the volume of the cerebellum belonged to the cortex and was evenly distributed between the molecular and granular layers (*p* = 0.68). Cerebellar nuclei were the smallest part of the cerebellum and only made up 3% of its total volume. The CE of the total volume of the cerebellum was 0.02 ± 0.003% (mean ± standard deviation).

The parameters of the microvascular bed are shown in Figures 2B–F and Figure 3B. No significant differences were found in the number of vessels between the whole cerebellum and the midbrain. Furthermore, the midbrain vessels were significantly shorter with greater *D*_*D*_. However, each of the cerebellar components had its own features of the microvascular bed (Figure 2C).

The largest *N* of the short microvessels was found in the granular layer. The *N* in the white matter and nuclei of the cerebellum were relatively lower than in the granular layer. Differences in *N*_*D*_ between the individual cerebellar structures were observed (Figure 2D).

Only the granular layer of the WT cerebellum had a corresponding *L* of vessels as the whole midbrain (Figure 2E). The *L* in the individual structures of the cerebellum were positively correlated with their *N* (Spearman *R* = 0.9). That is why the differences in *L* were similar as in *N*. Despite this, a higher *L*_*D*_ of vessels was found in the cerebellar nuclei and the granular layer. *L*_*D*_ was significantly lower in the molecular layer, and the lowest *L*_*D*_ was observed in the white matter (*p* = 0.04). Due to the vessels' density, their *D*_*D*_ in the granular layer and nuclei were shorter than in the molecular layer and white matter.

Based on the above observations, we rejected the H0(1) hypothesis.

### Comparison of cerebellar vascularity in WT and Lc mice

As expected, neither the volume of the midbrain nor the parameters characterizing the microvascular bed of the midbrain of Lc differed from those of the WT midbrain (Figure 2). The only exception was the mean length of the midbrain vessels that was significantly lower in Lc than in WT (Figure 3B).

**Figure 4:**
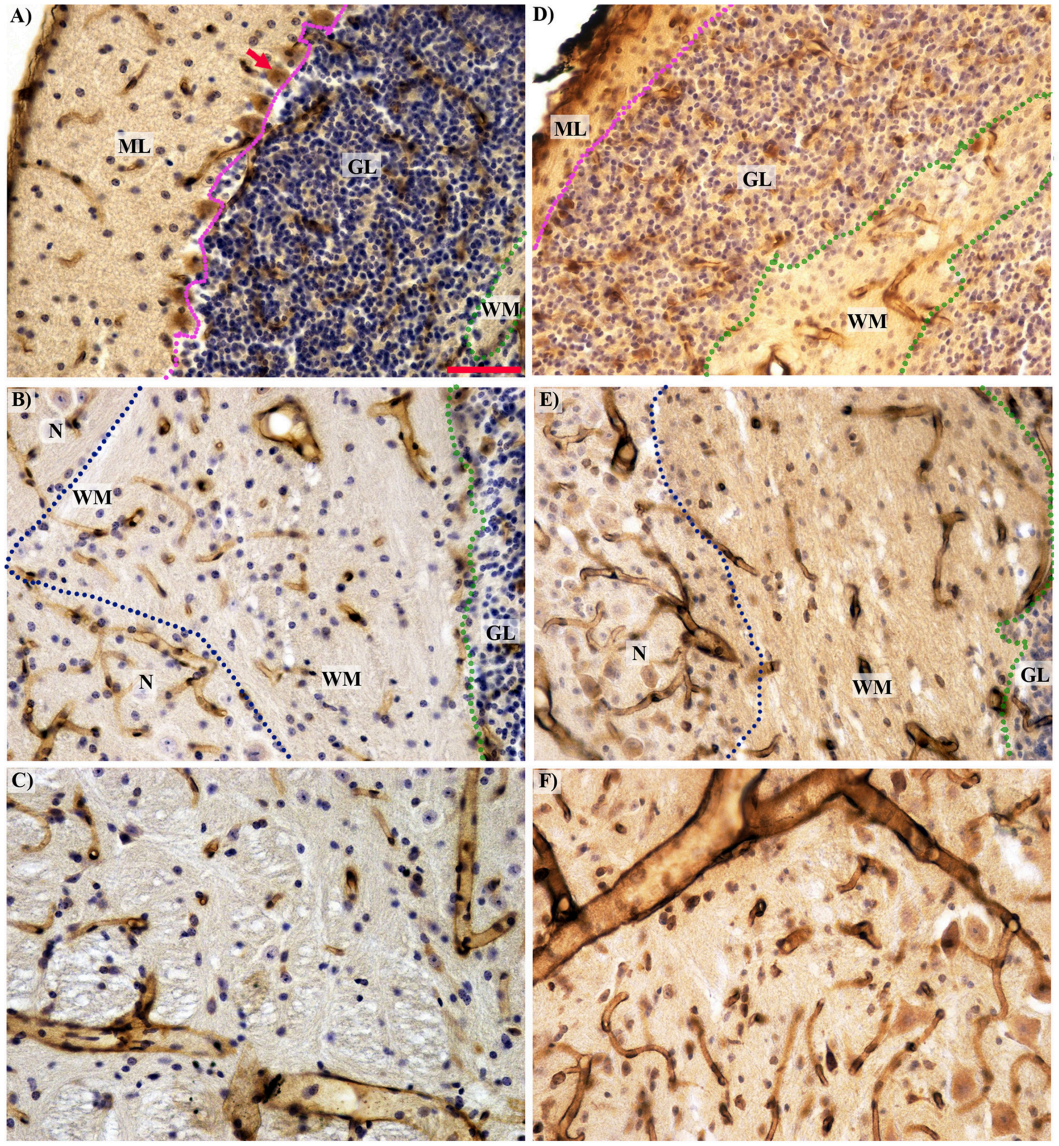
**Histological comparison of microcirculation in the separated cerebellar layers and the midbrain in the wild type (A–C) and Lurcher (D–F) mice**. **(A,D)**—The complete absence of Purkinje cells (red arrow) with intensive degeneration of the molecular (ML) and granular (GL) layers in Lurcher mice; **(B,E)**—Morphological features of vascularity in the white matter (WM) and nuclei (N). The boundary between the molecular and granular layers marked by the purple dotted lines; between the granular layer and white matter by the green and between the white matter and the nuclei by the blue dotted lines. **(C,D)**—No significant difference in the midbrain vascularity. Immunohistochemical detection of the microvessel laminin outlines, visualization with horseradish peroxidase/diaminobenzidine (brown), counterstaining with haematoxylin. Scale bar: 50 μm, uniform magnification in **(A–F)**.

The total volume of the Lc cerebellum was reduced more than three times compared with WT mice. The most apparent reductions in volume were observed in the cortex and were uniform throughout the separated layers of the cortex. The volume of white matter was also reduced, but not as drastically as the cortex layers. On the other hand, the cerebellar nuclei were the only cerebellar structures that were not significantly reduced in Lc (Figures 2A, 4). The Lc nuclei also showed significantly lower mean vessel length than WT, while this parameter remained unchanged in the rest of the cerebellum compared with WT (Figure 3B). The CE of the total volume of the cerebellum was 0.041 ± 0.002%.

With the reduction of the cerebellar volume, the vessel number was also reduced more than twice in the Lc mice (Figure 2C). Particularly, this process was most pronounced in the granular layer and was also detected in the molecular layer, while *N* remained unchanged compared with WT in the white matter and cerebellar nuclei.

The *N*_*D*_ per unit volume was increased in the total Lc cerebellum as well as in the individual cortex layers compared with WT. The *N*_*D*_ in the Lc cerebellar nuclei was non-significantly higher than in WT (Figure 2D). The *N*_*D*_ in the white matter was not different from that in WT (Figure 2D).

Reductions of *L* were also shown in the total Lc cerebellum and in individual cerebellar structures, except the nuclei (Figure 2E). Furthermore, in contrast to WT, *L* was significantly lower than in the midbrain (Figure 2E). No significant differences between *L* of the white matter and the cerebellar nuclei were found in the Lc mice.

The changes of *L*_*D*_ were found only in the molecular layer. Furthermore, the *L*_*D*_ in the molecular and granular layers were similar in the Lc cerebellum. The *D*_*D*_ of the molecular layer was also reduced. A similar trend was observed in the other layers of the Lc cerebellum.

The complete data set with all of the morphometric results for all of the samples is provided in Supplementary Table 1, and tables with the statistics reporting the values in details were included as Supplementary Table 2.

Relying on these arguments, we rejected the H0(2) and H0(1) hypotheses for the Lc mice.

### The correlations among morphometric characteristics in WT and Lc mice

The correlations between morphometric parameters for each of the experimental groups are presented separately in Table 2. In this experiment, only strong positive correlations between the parameters (Spearman *R* = 0.9) achieved statistical significance. The *V* of the total WT cerebellum was positively correlated with the midbrain volume. This relationship was lost in the cerebellar degeneration model. However, the correlation between *L* of the midbrain and total cerebellum *L* appeared in the Lc mice. The *V* of the molecular layer correlated with the white matter, the nuclei, and the midbrain volumes. Additionally, the sizes of the nuclei and white matter depended on the total cerebellar volume in the control animals. In contrast to WT, only the correlation between the molecular layer and white matter volumes was detected in the Lc mice.

**Table 2:** **Spearman rank order correlations between the quantitative parameters**.

		**Molecular layer and Purkinje cells**			**Granular layer**			**White matter**			**Nuclei**			**Total cerebellum**			**Midbrain**			
		***V***	***N***	***L***	***V***	***N***	***L***	***V***	***N***	***L***	***V***	***N***	***L***	***V***	***N***	***L***	***V***	***N***	***L***	
Molecular layer and Purkinje cells	*V*		–	–	–	–	–	**0.9**	**X**	–	–	–	–	–	–	**X**	–	–	–	Lurcher
	*N*	–		–	–	–	–	**X**	**0.9**	–	–	–	–	–	**X**	**X**	**X**	**0.9**	–	
	*L*	–	–		–	–	–	**X**	**X**	–	–	–	–	–	–	**X**	–	–	–	
Granular layer	*V*	–	–	**0.9**		–	–	–	–	–	–	–	**X**	–	–	–	–	–	–	
	*N*	–	**0.9**	**X**	–		–	–	–	–	–	–	**X**	–	–	–	–	–	–	
	*L*	–	–	–	–	–		**X**	**X**	–	–	–	–	–	–	**X**	–	–	–	
White matter	*V*	**0.9**	–	**0.9**	–	–	–		–	**0.9**	–	–	–	–	–	–	–	–	**X**	
	*N*	–	–	–	–	–	–	–		**X**	–	–	–	–	–	–	–	–	**X**	
	*L*	–	–	–	**X**	–	**0.9**	–	**X**		–	–	–	–	–	**X**	–	–	–	
Nuclei	*V*	**0.9**	**X**	–	–	–	–	–	–	–		–	**0.9**	–	–	–	–	–	–	
	*N*	**X**	**0.9**	–	–	–	–	–	–	–	–		**X**	–	**X**	–	**X**	**0.9**	–	
	*L*	–	–	–	–	**X**	–	–	–	–	**0.9**	**X**		**X**	–	–	–	–	–	
Total cerebellum	*V*	–	–	–	–	–	–	**X**	–	–	**X**	**X**	–		–	–	–	–	–	
	*N*	–	**X**	**X**	–	–	–	–	–	–	–	–	**X**	–		–	–	–	**X**	
	*L*	–	–	–	**X**	**X**	**X**	**X**	–	–	–	–	–	–	**X**		–	–	**0.9**	
Midbrain	*V*	**0.9**	**X**	–	–	–	–	–	–	–	–	–	**X**	**0.9**	–	–		–	**X**	
	*N*	–	–	–	–	–	–	–	–	–	–	–	–	–	–	–	–		**X**	
	*L*	**X**	**X**	–	–	–	–	–	–	–	–	–	**0.9**	**X**	–	–	–	–		
Wild type																				

*The data were pooled across the experimental groups, Lurcher—on a grey background and wild type (on a white background). V—volumes of analysed anatomical structures; N—total number of microvessels that were found in the corresponding structures and their total length. All correlations were considered significant at p < 0.05. The correlations without clear biological grounds are replaced by X. The remaining correlations have no statistical significance (p > 0.05) and are replaced by the—sign*.

The correlation between *N* in the nuclei and the total cerebellum volume was not observed in Lc the mice. Furthermore, the *N* in the molecular layer of the WT mice correlated with the *N* in the granular layer and the cerebellar nuclei. A new relationship between the *N* of the molecular layer and the *N* of the white matter or midbrain, which was not found in WT mice, was also observed in the Lc mice.

The correlations between *L* in the individual cerebellar structures in WT and Lc mice were different. Specifically, in the WT mice, the *L* in the granular layer correlated with the *L* in the white matter and the *L* in the nuclei correlated with the *L* in the midbrain.

## Discussion

### Vascularization in the cerebellum of healthy mice

The mouse cerebellar cortex takes up more than 80% of the total volume of the cerebellum. Approximately half of the cortex volume is taken up by the granular layer, while the other half is taken up by the molecular and the Purkinje cell layers. The largest number and the greatest length of blood vessels was found in the cerebellar cortex (Figures 2C,E). Furthermore, approximately half of all of the cerebellum microvessels are located in the granular layer (Figure 3). These vessels are relatively short and form a dense network. A high density of vessels and a short diffusion distance were also observed in the cerebellar nuclei. Neuron activity requires a large amount of nutrients (Rhyu et al., 2010) which is why the length of nuclei vessels is closely linked with the volume. Thus, it is not surprising that the granular layer and the cerebellar nuclei, which contain a large number of neurons, exhibited the highest density of the vessel beds. White matter activity is lower than in the gray matter; therefore, its vascularity is lower as well. As a consequence, the diffusion distance of the blood vessels is greater.

Unfortunately, studies that described in the details microcirculation in the human cerebellum are lacking. In particular, no studies have compared the cerebellum vascularization between humans and experimental animals. The only available data show that the *N*_*D*_ of vessels in different areas of the human cerebral cortex and in the deep nuclei are more than twice as high as in the gray matter of the mouse cerebellum, while the numerical density of vessels in the white matter in mice is more than six times greater (Tonar et al., 2011). The *L*_*D*_ in the mouse cerebellar cortex roughly corresponds to the *L*_*D*_ in the motor cortex of the rat (Tata and Anderson, 2002) and is almost half as much as in the human cerebral cortex (Cassot et al., 2006; Tonar et al., 2011). The same can be said about *L*_*D*_ in the subcortical gray matter. Conversely, the *L*_*D*_ of the vessels in the white matter of the mouse cerebellum is twice as much as in humans brain (Tonar et al., 2011).

Summarizing these data, it can be argued that the general trends in the vascularization of the human brain and mouse cerebellum are similar, despite the essential difference in the distribution of vessels between the gray and white matter. In addition, regardless of the much larger vessels in the human brain, differences in their diffusion distance can be expected only in the white matter. However, the rate of blood flow can be significantly different in these areas (van Raaij et al., 2012).

### Cerebellar vascularity of lurcher mice

In the three-month-old Lc mice, the total volume of the cerebellum was reduced to 71% compared with WT mice, mainly due to degeneration of the cortex. The cyto-architecture of the cerebellar cortex was disturbed, and the granular and molecular layers were poorly defined (Figures 4A,B).

The reduction of the molecular layer volume is caused by massive Purkinje cell degeneration and destruction of their dendrite communication, as well as degeneration of basket and stellate cells and reduction of the axons of the granule cells known as parallel fibers (Caddy and Biscoe, 1979; Caddy and Herrup, 1991; Zanjani et al., 2006). Therefore, each surviving Purkinje cell in the Lc/WT chimera accepts an increased supply of the afferent signals from the granule cells, and they are unable to process them (Caddy and Herrup, 1990). Extensive reduction of granule cell volume is due to secondary target-related death of approximately 90% of the granule cells (Wetts and Herrup, 1982a,b, 1983).

The disappearance of the Purkinje cell axons and the degeneration of the climbing fibers originating in the inferior olive (Caddy and Biscoe, 1979) explain the reduction of white matter volume with preservation of the correlation between the molecular layer and the white mater volume (Table 2). Because the axons of the cells occupy less space than their body, the intensity of reduction was less pronounced in the white matter volume (Figure 2A).

Heckroth (1994a,b) reported decreasing nuclei volume between 10 and 30 days of age in Lurcher mutants. This decrease was accompanied by a reduction in neuron number in certain nuclei and was explained as the effect of nuclei differentiation. Similar results have been observed previously (Sultan et al., 2002). In contrast, we did not observe any significant differences in the nuclei volume and their vascularity between adult Lurcher and healthy mice.

Many studies have shown that degeneration of the central nervous structures is accompanied by changes in their vascular bed. In some cases, these changes are only secondary, while in other cases they can participate in the pathogenesis of the neurodegenerative disorder (Kolinko et al., 2015). In Lurcher mice, we have shown that the cerebellar degeneration also involves vascular changes. With decreases of the cerebellum volume in Lurcher mice, the number and length density of microvessels (see Figures 2C,E) is reduced more than two-fold (to 53 and 65%, respectively) without a clear correlation between these processes. These changes were more apparent in the cerebellar cortex that undergoes massive degeneration but less apparent in the white matter and not observed cerebellar nuclei that were relatively spared.

Vascular changes were restricted to those cerebellar structures that undergo substantial degeneration, and the intensity of both processes revealed several correlations mostly expressed in the cerebellar nuclei and the white matter. This indicates a relationship between neural degeneration and vascular changes. Furthermore, we have shown that the midbrain of Lurcher mice did not exhibit any changes in volume, but some delicate changes in the vascular bed, such as reduction of the average vessel length, were observed. Therefore, it can be assumed that vascular changes in Lurcher mice are specific to degenerating cerebellar structures and have a comprehensive nature.

The reduction in the number of vessels in the cerebellar cortex can be explained in general by one of the two mechanisms. The first mechanism is the inhibition of angiogenesis during development due to the lower number of neurons (Swain et al., 2003). The latter mechanism is the regression of existing vessels due to the supersaturation of tissue from them (Rhyu et al., 2010). Currently, we do not have a clear answer to this question, but we are leaning toward to the first mechanism.

Because the reduction of the vessels was relatively milder than the reduction of the volume, the numerical densities of vessels per unit volume are increased. Thus, the increase of the vessel density could be explained by shrinkage of the tissue due to the extinction of the neurons and nerve fibers. Here, the diffusion distance of the cerebellar vessels in the Lurcher mice did not exhibit significant fluctuations compared with the WT mice. The higher density of the vessels and the shorter diffusion distance in the Lurcher mutant cerebellar cortex increases the capacity of blood delivery and nutrient supply. On the other hand, in the mutant cerebellum, a higher basal level of IL-1b expression was observed (Vernet-der Garabedian et al., 1998). This cytokine underlines the supportive role of the inflammatory reaction in the massive neurodegeneration process. Therefore, the increase in the vessel density can be associated with the increase in immune activity in this area.

A sharp increase in the *N*_*D*_ and *L*_*D*_ in the nucleus of the cerebellum of Lc mice is interesting, but statistically insignificant. In our opinion, it was due to approaching the vessels from the destructed white matter or due to the activation of angiogenesis as a protective mechanism to preserve individual vital functions (Swain et al., 2003; Rhyu et al., 2010).

Nevertheless, in general it remains unclear whether the relation between the neuronal degeneration and vascular changes is causal or not. We are convinced that vascular changes are not the primary factor starting the degeneration. Among the cerebellum, the GluRδ2 is specific for Purkinje cell bodies and dendrites (Araki et al., 1993) and there is no evidence to expect its expression on vessels. The *Grid2*^*L*^^*c*^ changes GluRδ2 into a leaky membrane channel that chronically depolarizes Purkinje cell membrane and thereby induces cell-autonomous death of these cerebellar cells (Zuo et al., 1997). The process of Purkinje cell death has been alternatively described as excitotoxic apoptosis, autophagy, necrosis, or combination of these proseccess (Norman et al., 1995; Zuo et al., 1997; Selimi et al., 2000; Yue et al., 2002; Dusart et al., 2006; Wang et al., 2006; Nishiyama and Yuzaki, 2010; Zanjani et al., 2013). Degeneration of the other cell types is supposed to be secondary (target-related or trans-synaptic degeneration) to Purkinje cell loss (Wetts and Herrup, 1982a,b). From this point of view it seems that vascular changes are rather secondary to the neurodegenerative process and thus even tertiary to the *Grid2*^*Lc*^ mutation. However, they can modify its progress. Theoretically it could be in both a positive as well as negative manner. As suggested above, higher vessel density increases the availability of the nutrients which are crucial for hyperpolarized Purkinje cells that have higher demand for energy (Nishiyama and Yuzaki, 2010). On the other hand, higher vascular density could increase exposure of the tissue to pro-inflammatory cytokines potentially enhancing the degenerative process (Vernet-der Garabedian et al., 1998) and increase vulnerability of the neurons threatened with the degeneration to other noxious factors (Caddy and Vozeh, 1997). To elucidate this question, a study of changes in microvasculature during ontogenesis showing the time relation between the loss of neurons and increase in vessel density has to be done in Lurcher mice.

### The practical value of the results

The cerebellar nuclei are the most spared structure of the Lurcher mutant cerebellum. The neurons of the deep cerebellar nuclei are involved in the modulation and proper performance of the ongoing conditioned responses that initiate learning processes (Gruart et al., 1997; Delgado-García and Gruart, 2002; Jiménez-Díaz et al., 2004). In fact, the cerebellar cortex, the deep cerebellar nuclei, and/or a combination of them with extra-cerebellar structures are responsible for motor learning (Porras-García et al., 2013). Moreover, the cerebellar cortex is involved in fear conditioning and anxiety and modulates fear-related behaviors (Lorivel et al., 2014). This means that any injuries to the nuclei of the cerebellum lead to deterioration of learning ability (Porras-Garcia et al., 2010) and innate fear-related behaviors (Lorivel et al., 2014). This is why the preservation of the cerebellar nuclei is vitally important. Furthermore, when the cortex degenerates, the nuclei should be more active due to the loss of inhibitory Purkinje cell projection. Long-term loading usually leads to an increase in vascular volume fraction (Swain et al., 2003; Rhyu et al., 2010). Because the Purkinje cells, which are virtually completely missing in adult Lurcher mice (Caddy and Biscoe, 1979), represent the sole output from the cerebellar cortex, Lurcher mice can be considered a model of functional cerebellar decortication (Porras-Garcia et al., 2005). Under these conditions, cerebellar nuclei are the only structures that are likely to be responsible for persisting cerebellar functions in Lurcher mice. For instance, Lurcher mice exhibit some capability for classical conditioning, despite changes in the execution of the conditioned eyelid response (Porras-Garcia et al., 2005, 2010). These findings provide the evidence for that in the cerebellum; the cerebellar nuclei are the most important structure for classical conditioning and for control of kinematics of the eyelid response (Gruart et al., 1997; Delgado-García and Gruart, 2002; Jiménez-Díaz et al., 2004; Sánchez-Campusano et al., 2012).

Neurotransplantation is a potential therapy for cerebellar degeneration (Björklund and Lindvall, 2000). Its functional effect was explained by the reconstruction of the neural circuitries (Sotelo and Alvarado-Mallart, 1987). However, neurotransplantation still needs in-depth investigation before it can become a routine method in humans (Cendelin et al., 2009; Babuska et al., 2015; Cendelin, 2016).

Because the cerebellar nuclei had the shortest diffusion distance providing the best accessibility of nutrients in wild type as well as Lurcher mice, they could be relatively permissive structures for neural grafts in Lurcher mice. This location is also the most suitable for graft position because it is associated with the maturation of Purkinje cells during prenatal development (Machold and Fishell, 2005; Fink et al., 2006). Moreover, the proximity of the grafted Purkinje cells to the deep cerebellar nuclei is necessary for the establishment of synaptic contacts between grafted Purkinje cells and the host cerebellar nuclei (Keep and Sotelo, 1992) because the granular layer acts as a barrier that prevents nerve fibers from sprouting toward the deep cerebellar nuclei (Carletti et al., 2008). Transplantation of various types of stem cells also led to the improvement of motor function (Jones et al., 2010). However, induced pluripotent and mesenchymal stem cells would potentially elude the immune system or promote the regeneration of trophic factors (Jones et al., 2010; Ruff and Fehlings, 2010). Therefore, investigation within this research field should continue. Density assessment of the different cell types within the histological layers of the cerebellum and analysis of the condition of the neurovascular unit (blood-brain barrier) subcomponents during neurodegeneration could serve as further continuation of our research. It would also be interesting to investigate potential sex and age differences in the cerebellar microvasculature and map their distributions within anatomical lobules of the cerebellum.

## Conclusion

We determined that each of the cerebellar structures has its own features of vascularity. The shortest diffusion distance related to the highest density of microvessels was found in the cerebellar nuclei and in the granular layer in both wild type and Lurcher mutant mice. These structures are characterized by high cell density compared with the molecular layer and white matter, thus, the character of their vascular bed corresponds with the high demand for blood supply.

We have confirmed the severe reduction of the volume of the Lurcher mutant cerebellum and its granule and molecular layers (Caddy and Biscoe, 1979; Baurle et al., 2006). Reduction of the white matter can be explained by the extinction of Purkinje cell axons and the reduction of the climbing fibers (Caddy and Biscoe, 1979). On the other hand, reduction of cerebellar nuclei volume was not confirmed (Heckroth, 1994a,b; Sultan et al., 2002).

Cerebellar degeneration was accompanied with marked changes of the microvascularization in Lurcher mutants. Nevertheless, the reduction of the capillary bed was milder than the decrease in the cerebellar volume, and therefore the density of the vessels became higher in the cerebellar cortex of the Lurcher mutants. In the cerebellar nuclei, no changes of the capillary bed were observed. This suggests that the vascular changes were restricted to cerebellar structures affected by the degeneration and are related to the shrinkage of the volume of the individual cerebellum layers. Furthermore, the vasculature of the midbrain was also affected in the Lurcher mice.

The increase of the capillary density in the Lurcher cerebellar cortex might increase the availability of nutrients and oxygen as well as the delivery of pro-inflammatory cytokines other regulatory factors or noxious substances. These factors influence the niche of the cerebellar cortex of Lurcher mice. These factors could potentially play some minor role in the pathogenesis of the degeneration or in secondary changes to the tissue and could also have an essential impact on regenerative or neurotransplantation therapies for cerebellar degeneration.

## Author contributions

KY performed the histological morphometry, the data analysis, the literature searches and wrote the manuscript; CJ provided the biological material, contributed to the generation of ideas, general editing, and commenting on the text; KM contributed to the generation of ideas, general editing, and commenting on the text; TZ offered the methodology for vascular stereological assessment, contributed to the generation of ideas, general editing, and commenting on the text.

## Funding

This study was supported by the National Sustainability Program I (NPU I) Nr. LO1503 provided by the Ministry of Education Youth and Sports of the Czech Republic.

### Conflict of interest statement

The authors declare that the research was conducted in the absence of any commercial or financial relationships that could be construed as a potential conflict of interest.

## Supplementary material

Supplementary Table 1 **The primary data of the stereological assessment of cerebellar vascularity in the wild type (WT) and Lurcher (Lc) mice**.

Supplementary Table 2 **Statistical evaluation of reported values when comparing wild type and Lurcher mice. (A)** Mann–Whitney U-test to compare both animal groups; **(B)** Wilcoxon signed-rank test for comparing the data within each group.
