# Supplementary material for: Smaller Absolute Quantities but Greater Relative Densities of Microvessels Are Associated with Cerebellar Degeneration in Lurcher Mice
Source: Front Neuroanat. 2016 Apr 19;10:35. doi: 10.3389/fnana.2016.00035 (PMC4835681; doi:10.3389/fnana.2016.00035)
Supplement: Supplementary Table 2 — Statistical evaluation of reported values when comparing wild type and Lurcher mice. (A) Mann–Whitney U-test to compare both animal groups; (B) Wilcoxon signed-rank test for comparing the data within each group. [file Table2.pdf]

A) Mann-Whitnny u test

WT/Lc	Volume (mm^3)	Number of vassels	Length density of vassels (mm)	Number of vessels in 1 mm^3	Length density of vassel in 1 mm^3 (mm)	Mean length of vessel (mm)	Diffusion distance (mm)
Molecular layer	0.012	0.012	0.012	0.037	0.022		0.022
Granular layer	0.012	0.012	0.012	0.012			
White matter	0.012		0.012				
Nuclei						0.037	
Cerebellum	0.012	0.022	0.012	0.012			
Midbrain						0.012	

B) Wilcoxon signed-rank test

Volume

	Molecular layer	Granular layer	White matter	Nuclei	Cerebellum	Midbrain	
Molecular layer		0.043	0.043	0.043	0.043	0.043	
Granular layer	0.043			0.043	0.043	0.043	
White matter		0.043		0.043	0.043	0.043	Lurcher
Nuclei	0.043	0.043	0.043		0.043	0.043	
Cerebellum	0.043	0.043	0.043	0.043		0.043	
Midbrain	0.043	0.043	0.043	0.043	0.043		

Wild type

Number of vassels

	Molecular layer	Granular layer	White matter	Nuclei	Cerebellum	Midbrain	
Molecular layer		0.043		0.043	0.043	0.043	
Granular layer	0.043		0.043	0.043	0.043	0.043	
White matter	0.043	0.043			0.043	0.043	Lurcher
Nuclei	0.043	0.043	0.043		0.043	0.043	
Cerebellum	0.043	0.043	0.043	0.043		0.043	
Midbrain		0.043	0.043	0.043			

Wild type

Length density of vassels

	Molecular layer	Granular layer	White matter	Nuclei	Cerebellum	Midbrain	
Molecular layer		0.043	0.043	0.043	0.043	0.043	
Granular layer	0.043		0.043	0.043	0.043	0.043	
White matter	0.043	0.043			0.043	0.043	Lurcher
Nuclei	0.043	0.043	0.043		0.043	0.043	
Cerebellum	0.043	0.043	0.043	0.043		0.043	
Midbrain	0.043		0.043	0.043	0.043		

Wild type

Number of vassels in1 mm^2

	Molecular layer	Granular layer	White matter	Nuclei	Cerebellum	Midbrain	
Molecular layer			0.043	0.043			Lurcher
Granular layer	0.043		0.043		0.043	0.043	
White matter		0.043		0.043	0.043		
Nuclei			0.043		0.043	0.043	
Cerebellum	0.043	0.043	0.043			0.043	
Midbrain			0.043				
	Wild type						

Length density of vassel in 1 mm^3

	Molecular layer	Granular layer	White matter	Nuclei	Cerebellum	Midbrain	
Molecular layer			0.043		0.043	0.043	Lurcher
Granular layer	0.043		0.043		0.043	0.043	
White matter	0.043	0.043		0.043	0.043		
Nuclei	0.043		0.043		0.043	0.043	
Cerebellum	0.043	0.043	0.043	0.043		0.043	
Midbrain		0.043		0.043	0.043		
	Wild type						

Diffusion distance of vessel

	Molecular layer	Granular layer	White matter	Nuclei	Cerebellum	Midbrain	
Molecular layer			0.005	0.012		0.028	Lurcher
Granular layer			0.005		0.005	0.005	
White matter	0.005	0.005		0.005	0.005		
Nuclei	0.012		0.005		0.005	0.005	
Cerebellum		0.005	0.005	0.005		0.005	
Midbrain	0.028	0.005		0.005	0.005		
	Wild type						

Mean length of vessel

	Molecular layer	Granular layer	White matter	Nuclei	Cerebellum	Midbrain	
Molecular layer				0.043	0.043	0.043	Lurcher
Granular layer	0.043		0.043				
White matter		0.043		0.043		0.043	
Nuclei					0.043	0.043	
Cerebellum		0.043					
Midbrain	0.043	0.043	0.043	0.043	0.043		
	Wild type						
